# Identification and cross-validation of autophagy-related genes in cardioembolic stroke

**DOI:** 10.3389/fneur.2023.1097623

**Published:** 2023-05-25

**Authors:** Yufang Yang, Min Zhang, Ziqing Li, Shen He, Xueqi Ren, Linmei Wang, Zhifei Wang, Shi Shu

**Affiliations:** ^1^School of Basic Medical Sciences, Shanghai University of Traditional Chinese Medicine, Shanghai, China; ^2^Division of Mood Disorders, Shanghai Mental Health Center, Shanghai Jiao Tong University School of Medicine, Shanghai, China

**Keywords:** autophagy, cardioembolic stroke, bioinformatics analysis, gene expression omnibus dataset, hub genes

## Abstract

**Objective:**

Cardioembolic stroke (CE stroke, also known as cardiogenic cerebral embolism, CCE) has the highest recurrence rate and fatality rate among all subtypes of ischemic stroke, the pathogenesis of which was unclear. Autophagy plays an essential role in the development of CE stroke. We aim to identify the potential autophagy-related molecular markers of CE stroke and uncover the potential therapeutic targets through bioinformatics analysis.

**Methods:**

The mRNA expression profile dataset GSE58294 was obtained from the GEO database. The potential autophagy-related differentially expressed (DE) genes of CE stroke were screened by R software. Protein–protein interactions (PPIs), correlation analysis, and gene ontology (GO) enrichment analysis were applied to the autophagy-related DE genes. GSE66724, GSE41177, and GSE22255 were introduced for the verification of the autophagy-related DE genes in CE stroke, and the differences in values were re-calculated by Student’s *t*-test.

**Results:**

A total of 41 autophagy-related DE genes (37 upregulated genes and four downregulated genes) were identified between 23 cardioembolic stroke patients (≤3 h, prior to treatment) and 23 healthy controls. The KEGG and GO enrichment analysis of autophagy-related DE genes indicated several enriched terms related to autophagy, apoptosis, and ER stress. The PPI results demonstrated the interactions between these autophagy-related genes. Moreover, several hub genes, especially for CE stroke, were identified and re-calculated by Student’s *t*-test.

**Conclusion:**

We identified 41 potential autophagy-related genes associated with CE stroke through bioinformatics analysis. SERPINA1, WDFY3, ERN1, RHEB, and BCL2L1 were identified as the most significant DE genes that may affect the development of CE stroke by regulating autophagy. CXCR4 was identified as a hub gene of all types of strokes. ARNT, MAPK1, ATG12, ATG16L2, ATG2B, and BECN1 were identified as particular hub genes for CE stroke. These results may provide insight into the role of autophagy in CE stroke and contribute to the discovery of potential therapeutic targets for CE stroke treatment.

## Introduction

Stroke constitutes the second leading cause of death and a major cause of disability worldwide (1). Cardioembolic stroke (also known as cardiogenic cerebral embolism, CE stroke) is one of the important complications of heart disease. In this case, due to various causes, the intracardiac mural thrombus sheds and flows into the cerebral artery then blocks the blood vessels, thus disrupting the compensation of the cerebral circulation and leading to the cerebral infarction. Atrial fibrillation remains the most common and studied mechanism underlying CE stroke events. The other pathological mechanisms associated with CE stroke include valvular disease, left ventricular dysfunction, and patent foramen ovale (2). Cardioembolic (CE) stroke constitutes approximately 20% of all occurrences of ischemic stroke in patients, with the highest recurrence rate and fatality rate. However, the omission diagnostic rate of CE stroke was as high as 10%–15% (3–5). Since oral anticoagulation is generally indicated for CE strokes to prevent recurrent events (6), the distinction between CE and non-CE strokes has important implications for clinical management.

As a critically regulated catabolic process, autophagy degrades cytoplasmic materials and provides substrates for energy metabolism during nutrient deprivation and metabolic stress (7). Insufficient supply of oxygen and glucose in cerebral ischemia leads to the activation of the AMPK pathway thus initiating autophagy. The role of autophagy has been studied in ischemic stroke for long and comes out to be extremely important and complicated for its two-edged contribution to neuronal death. Autophagy participates in the pathogenesis of ischemic stroke with various molecules involved (8). For instance, serum CXCL12 was positively related to stroke recurrence in a Chinese cohort diagnosed with acute ischemic stroke (9). The inhibition of hypoxia-inducible transcription factor alpha (HIF-1alpha) attenuated the activation of astrocyte formation in the ischemic animal (10). YTH domain-containing 1 (YTHDC1), one of the m (6)A readers, mitigated ischemic stroke through PTEN-mediated AKT phosphorylation (11). LncRNA PEG11as alleviates cerebral ischemia stroke via regulating autophagy both *in vivo* and *in vitro* (12). Autophagy was also involved in cardiogenic diseases. Autophagy-related genes, such as CTSB, ITGB1, CXCR4, and TNFSF10 were identified as potential diagnostic biomarkers for atherosclerosis (13). The key autophagy-related gene7 (ATG7) was significantly upregulated in AF patients, and autophagy induces atrial electrical remodeling via ubiquitin-dependent selective degradation of Cav1.2 (14). Six autophagy-related genes (BECN1, GAPDH, ATG7, MAPK3, BCL2L1, and MYC) were identified as significant potential regulators in valvular atrial fibrillation (15). In contrast, the autophagy-related pathogenesis of CE stroke has been barely studied. Therefore, the specific role of autophagy in CE stroke remains largely unknown, in which the autophagy-related hub genes remain to be revealed.

In this study, The GO datasets GSE58294 (16) and GSE66724 (17) that collected the blood samples of CE stroke patients and their matched controls were analyzed in the autophagy-related gene hub. Moreover, GSE41177 (18) and GSE22255 (19) datasets were taken cross-over analysis with the CE stroke datasets. GSE41177 consists of samples from the atrial fibrillation (AF) patients and the controls, while GSE22255 includes samples of generalized ischemic stroke patients and controls. The autophagy-related DE genes of CE stroke samples were explored. The protein–protein interactions (PPIs), correlation analysis, gene ontology (GO) enrichment analysis, and Kyoto Encyclopedia of Genes and Genomes (KEGG) pathway enrichment analysis were applied for the autophagy-related DE genes. Finally, the autophagy-related DE genes in CE stroke were verified in the AF dataset and generalized IS dataset. Therefore, the autophagy-related hub genes specialized for CE stroke were identified.

## Materials and methods

### Autophagy-related genes datasets and microarray data

A total of 222 genes were obtained from The Human Autophagy Database.^1^ The mRNA expression profile dataset of GSE58294 was downloaded from GEO.^2^ GSE58294 includes 23 cardioembolic strokes (≤3 h, prior to treatment) and 23 normal controls. GSE66724 includes 16 non-valvular AF patients of which eight had suffered a cardioembolic stroke between 2 and 11 years previously. GSE41177 includes 16 patients with AF and three patients with SR undergoing VHD surgery. GSE22255 includes 20 IS patients and 20 sex- and age-matched controls. All datasets are conducted in the GPL570 platform ([HG-U133 Plus 2] Affymetrix Human Genome U133 Plus 2.0 Array). Detailed information about the samples used in the GEO datasets is listed in Supplementary Table S7.

### Analysis of autophagy-related DE genes

The normalized expression matrix of microarray data was downloaded from the GSE58294, GSE66724, GSE41177, and GSE22255 datasets. Then the probes were annotated with the annotation files from the dataset. The “limma” package of R software was used to identify the autophagy-related DE genes. Genes with an adjusted *p*-value of 0.01 were considered DE genes. The heatmap, volcano plot, and box plot were conducted using the “heatmap” and “ggplot2” packages of R software.

### PPI analysis and correlation analysis of the autophagy-related DE genes

Protein–protein interaction analysis of autophagy-related DE genes was analyzed using the STRING database^3^ and Cytoscape software (version 3.8.1). The correlation analysis of the autophagy-related DE genes was identified using Spearman correlation in the “corrplot” package of R software.

### Go and KEGG pathway enrichment analysis of autophagy-related genes

Gene ontology and KEGG pathway enrichment analysis were conducted in R software using the package “GO plot.” The GO analysis consisted of cellular component (CC), biological process (BP), and molecular function (MF).

### Statistical analysis

The statistical analyses were performed using R software (version 3.6.3). Gene expression levels were re-calculated and compared using Student’s *t*-test. A *p*-value of <0.05 was considered statistically significant.

## Results

### Retrospective analysis of autophagy-related genes DE in cardioembolic stroke

**Figure 1:**
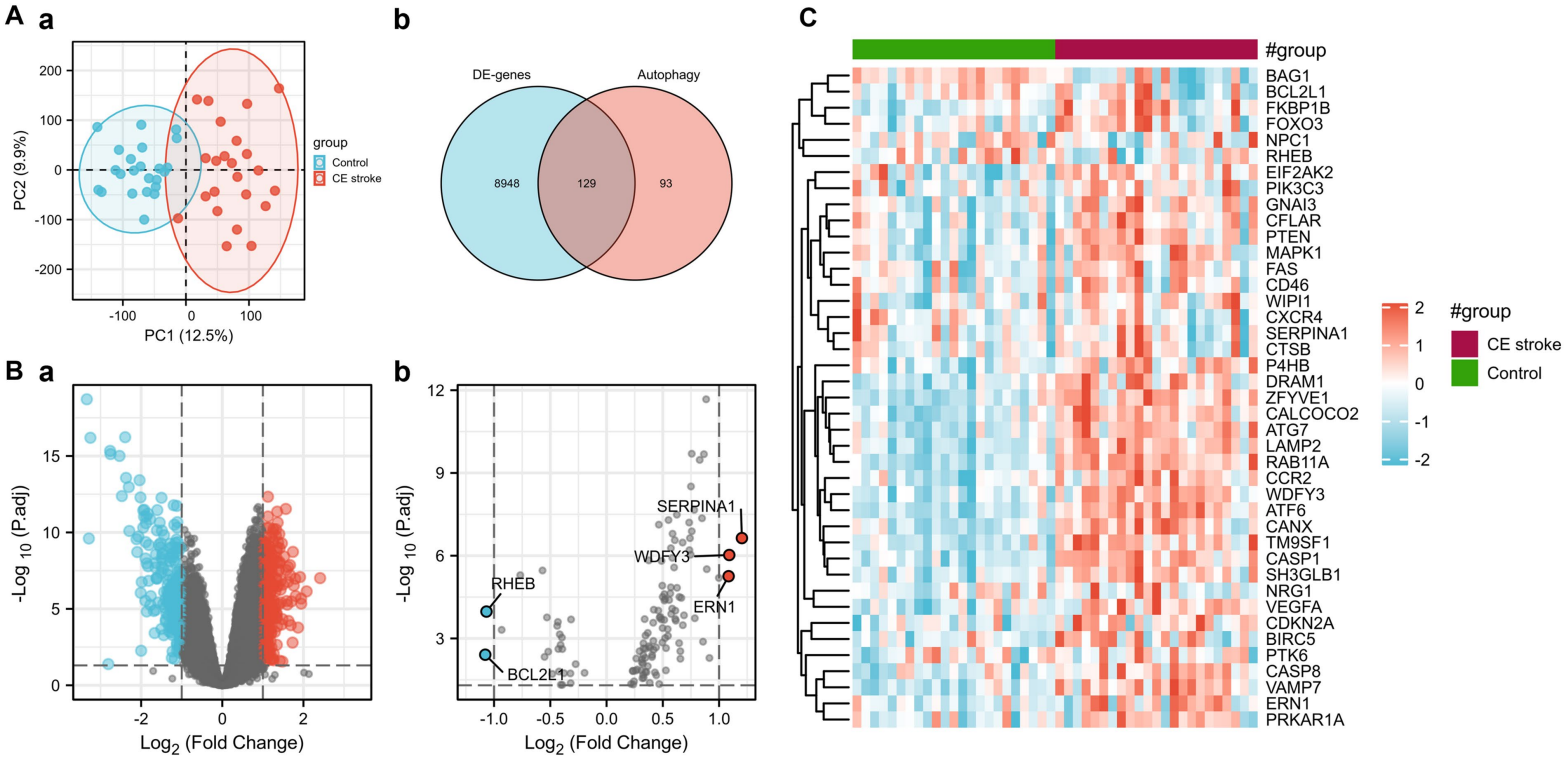
Differentially expressed autophagy-related genes in CE stroke and normal blood samples. **(A) (a)** Principal component analysis. **(b)** The Venn diagram of identifying differentially expressed genes with GSE58294 and autophagy-related genes. **(B) (a)** Volcano plot of differentially expressed genes of GSE58294 (logFC > 1, *p* < 0.01). **(b)** Volcano plot of the 129 differentially expressed autophagy-related genes (logFC > 1, *p* < 0.01). The red dots represent the significantly upregulated genes, and the blue dots indicate the significantly downregulated genes. **(C)** Heatmap of the 41 differentially expressed autophagy-related genes in CE stroke and normal blood samples.

**Figure 2:**
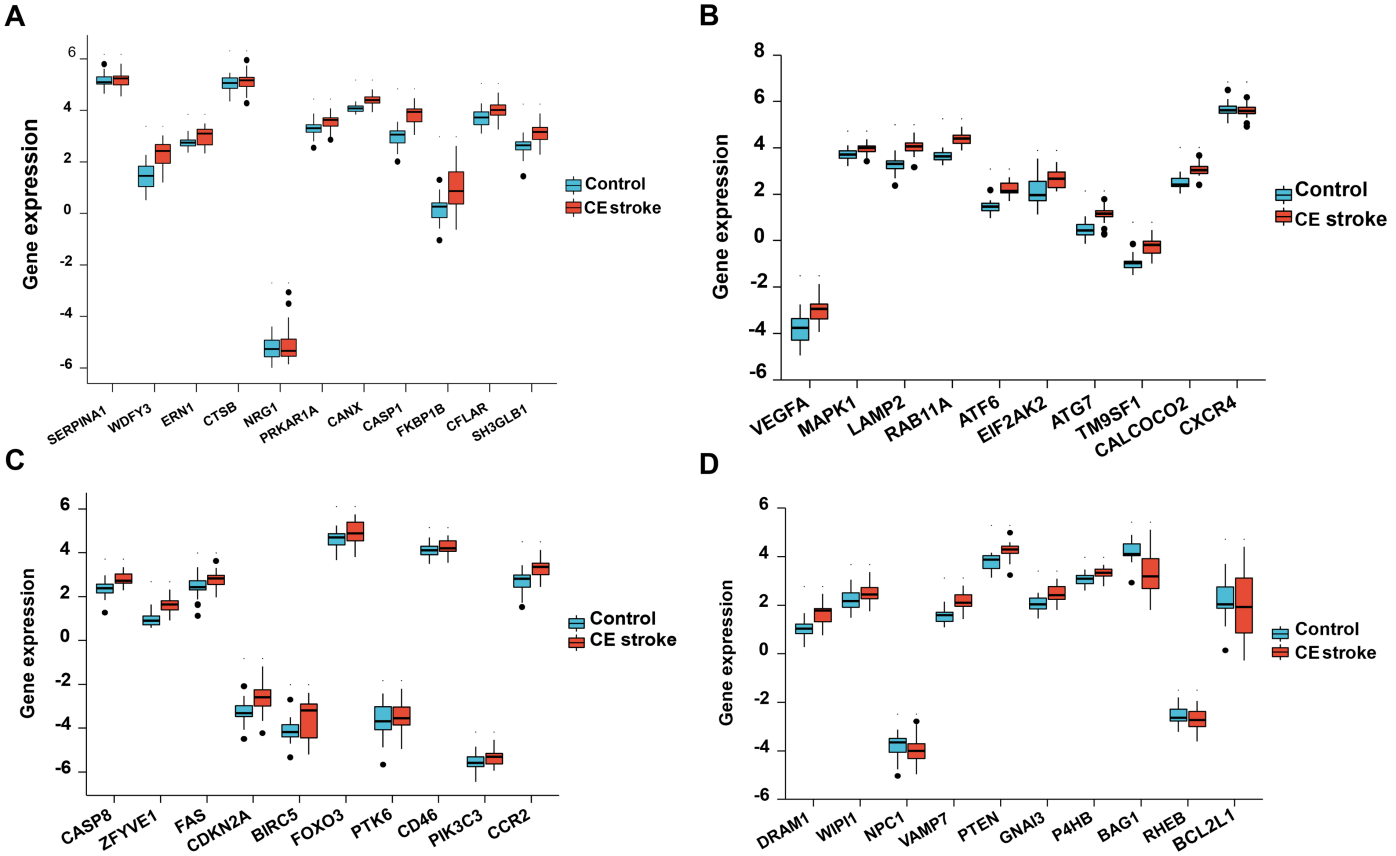
Boxplot of 41 differentially expressed autophagy-related genes in CE stroke and normal samples. The expression levels of each 11 differentially expressed autophagy-related genes are shown in **(A–D)**.

Principal component analysis (PCA) was performed to assess the reproducibility of the intra-group data of GSE58294 and it turned out to be fine (Figures 1Aa). Following the analysis of the GSE58294 dataset with R software, the whole DE gene set between the 23 CE stroke patients (≤3 h, prior to treatment) and 23 healthy individuals was compared with the 222 autophagy-related genes and 129 autophagy-related DE genes between CE stroke and normal groups were identified (Figures 1Ab; Supplementary Table S1). Then 41 autophagy-related genes were identified using the criteria of an adjusted *p*-value of 0.01 and an absolute fold-change value of >1.5, including 37 upregulated genes and four downregulated genes, which were presented with heatmap (Table 1; Figure 1C). The DE genes between CE stroke patients and normal controls in GSE58294 (logFC >1, *p* < 0.01) were shown with the volcano plot (Figures 1Ba). In this cohort, 129 DE genes were involved in autophagy, and the most differentially regulated genes were identified (logFC > 1, *p* < 0.01): the upregulated SERPINA1, WDFY3, and ERN1; the downregulated RHEB and BCL2L1 (Figures 1Bb). Moreover, the box plots showed the expression patterns of 41 autophagy-related DE genes between CE stroke and normal samples (Figures 2A–D).

**Table 1:** Forty-one differentially expressed autophagy-related genes in STROKE samples compared to healthy samples based on GSE58294.

DE-genes AND Autophagy	*p*-value	adj. *p*-value	logFC	FC	Absolute-logFC	Absolute-FC
SERPINA1	3.58E-09	2.3E-07	1.202835	2.301916	1.2028353	2.301916178
WDFY3	2.14E-08	9.44E-07	1.090347	2.129252	1.09034666	2.129251934
ERN1	0.000000189	5.54E-06	1.084768	2.121034	1.08476807	2.121034474
CTSB	0.000000226	6.38E-06	0.998078	1.997337	0.99807783	1.997337081
NRG1	0.000785	0.00511	0.914218	1.884547	0.91421804	1.884547345
PRKAR1A	9.17E-08	3.08E-06	0.89067	1.854037	0.89066983	1.854036736
CANX	1.52E-15	2.13E-12	0.884937	1.846683	0.88493652	1.846683364
CASP1	4.62E-13	2.09E-10	0.865293	1.82171	0.86529304	1.821709655
FKBP1B	0.000149	0.00131	0.857237	1.811566	0.85723705	1.811565601
CFLAR	4.23E-10	4.39E-08	0.848335	1.800422	0.84833478	1.800421602
SH3GLB1	8.49E-13	3.36E-10	0.827647	1.774788	0.82764696	1.774788315
VEGFA	0.0000137	0.000185	0.784238	1.722183	0.78423826	1.72218277
MAPK1	1.76E-10	2.18E-08	0.779045	1.715994	0.77904478	1.715994323
LAMP2	1.72E-09	1.3E-07	0.75699	1.689961	0.75698957	1.689960551
RAB11A	4.22E-13	2.01E-10	0.756858	1.689806	0.75685783	1.689806239
ATF6	1.52E-11	3.09E-09	0.750125	1.681938	0.75012478	1.681938297
EIF2AK2	2.07E-08	9.17E-07	0.747335	1.678689	0.74733478	1.678688772
ATG7	3.61E-09	2.31E-07	0.741006	1.671341	0.74100608	1.671340961
TM9SF1	1.27E-08	6.29E-07	0.736385	1.665996	0.73638451	1.665995507
CALCOCO2	6.71E-10	6.27E-08	0.734234	1.663514	0.7342338	1.66351376
CXCR4	0.0000102	0.000145	0.68805	1.611105	0.68805043	1.611104898
CASP8	0.0000281	0.00033	0.67762	1.599499	0.67761983	1.599498711
ZFYVE1	5.74E-09	3.33E-07	0.675777	1.597457	0.67577709	1.597456992
FAS	0.00000173	0.0000339	0.664528	1.58505	0.66452809	1.585049714
CDKN2A	0.000854	0.00545	0.656151	1.575873	0.65615087	1.575872561
BIRC5	0.000392	0.0029	0.655533	1.575198	0.65553302	1.57519782
FOXO3	0.00000157	0.0000313	0.647386	1.566328	0.64738609	1.566327713
PTK6	0.000172	0.00148	0.644899	1.563629	0.6448987	1.563629491
CD46	0.000000624	0.0000146	0.625929	1.543204	0.62592897	1.543204195
PIK3C3	2.89E-10	3.25E-08	0.624493	1.541669	0.62449304	1.541668991
CCR2	0.0000178	0.000227	0.615308	1.531885	0.61530783	1.531884823
DRAM1	0.00000636	0.000098	0.61167	1.528027	0.61166991	1.528026867
WIPI1	0.0000149	0.000197	0.610869	1.527179	0.61086927	1.527179106
NPC1	4.49E-09	2.74E-07	0.606458	1.522516	0.60645783	1.522516465
VAMP7	7.59E-08	2.67E-06	0.60045	1.516189	0.60044957	1.516188965
PTEN	2.24E-09	1.6E-07	0.586539	1.50164	0.58653913	1.50164015
GNAI3	2.85E-08	0.0000012	0.58504	1.500081	0.58504043	1.500081027
P4HB	0.000000169	5.08E-06	−0.76685	0.587698	0.76685404	1.701555301
BAG1	0.0000449	0.000488	−0.93241	0.523983	0.93240826	1.908459089
RHEB	0.00000695	0.000106	−1.06744	0.477166	1.06743739	2.095707525
BCL2L1	0.000562	0.00389	−1.07816	0.473631	1.0781644	2.111348017

### PPI network and correlation analysis of the DE autophagy-related genes of GSE58294

**Figure 3:**
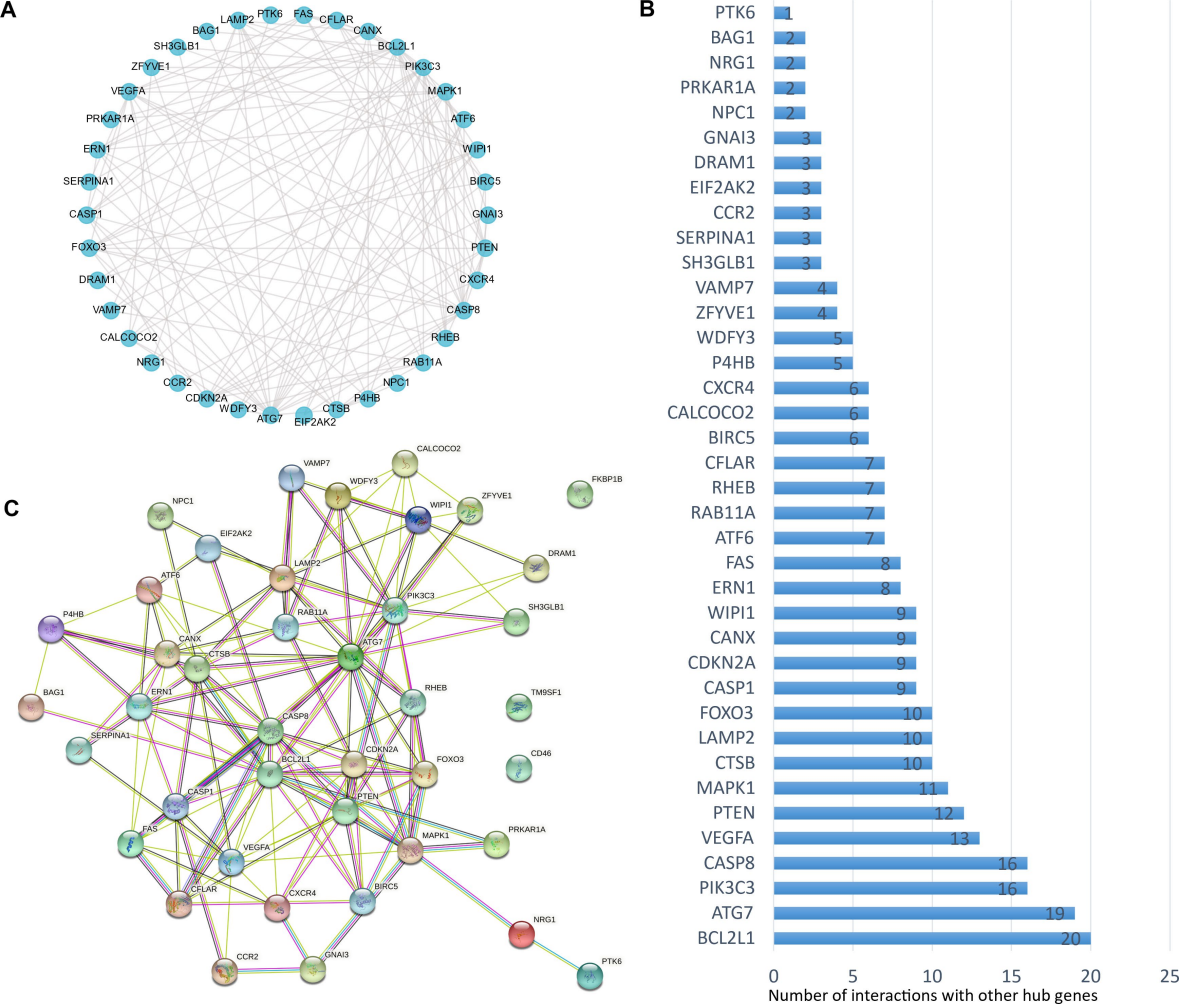
Protein–protein interactions (PPIs) analysis of the 41 differentially expressed autophagy-related genes. **(A)** The planimetric map of protein–protein interactions between the 41 differentially expressed autophagy-related genes. **(B)** The number of interactions between each individual gene and the other DE autophagy-related genes. **(C)** The 3D graphic model of PPI between the 41 differentially expressed autophagy-related genes. For known interactions, the lake blue line indicates that it is from curated databases, the purple line indicates that it is experimentally determined; for predicted interactions, the grass green line indicates gene neighborhood, the red line indicates gene fusions, the deep blue line indicates gene co-occurrence; for other types of interactions, the yellow line indicates text mining, the black line indicates co-expression, and the light blue line indicates protein homology.

**Figure 4:**
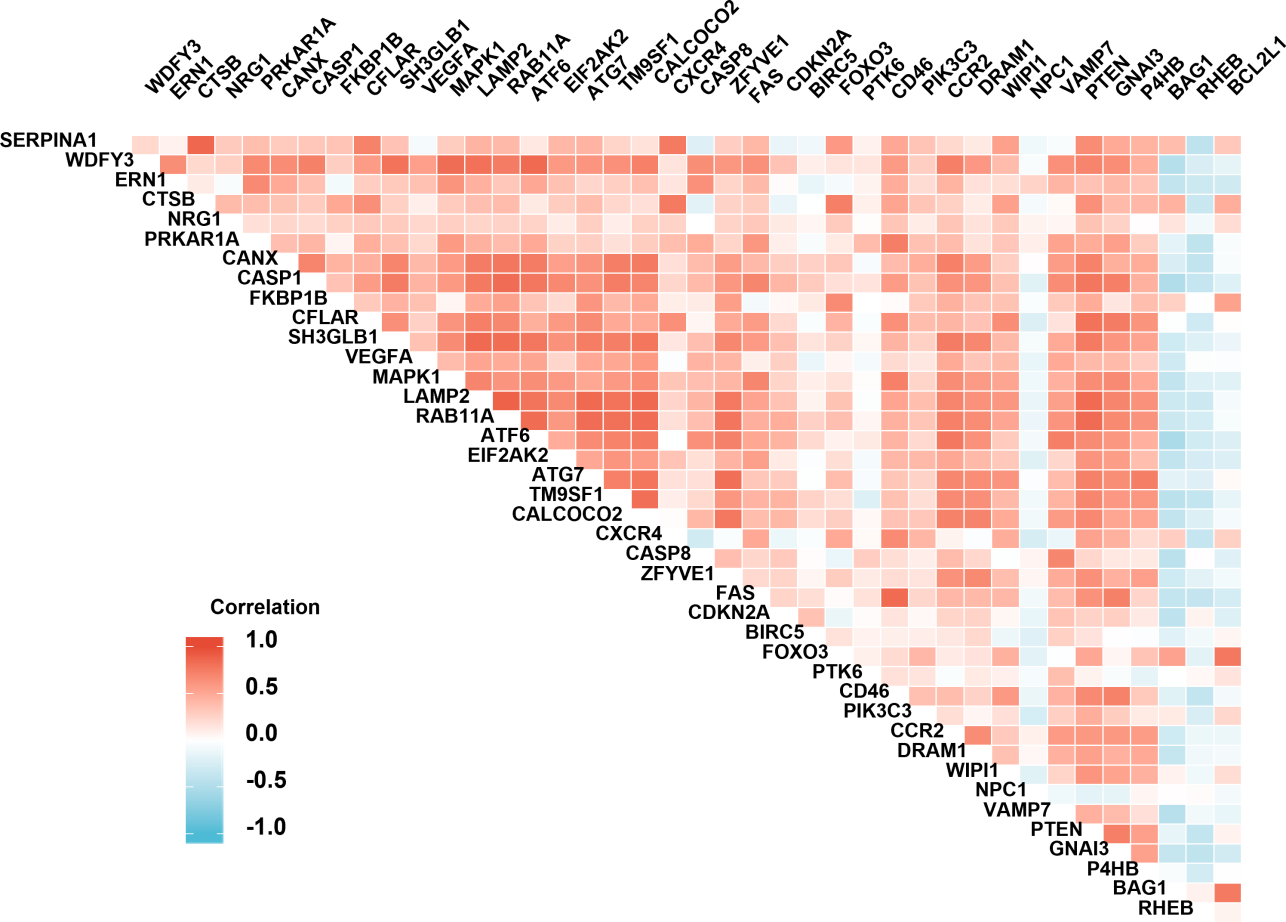
Spearman correlation analysis of the differentially expressed autophagy-related genes. The correlation scores (−1.0~+1.0) between every two individual genes were indicated with a red (positive correlation) or blue (negative correlation) lump.

The analysis of protein–protein interaction (PPI) for the autophagy-related DE genes was performed to establish the network and correlation among genes in the STRING database. The proteins encoded by these autophagy-related genes interact with each other (Figure 3A), and the number of interactions with each gene was counted (Figure 3B). With the 3D vision of the PPI network, the interacting nodes between genes were shown. Moreover, the correlation index between two genes including neighborhood_on_chromosome, gene fusion, phylogenetic co-occurrence, co-expression, experimentally determined interaction, automated text mining, and the combined score was presented (Figure 3C; Supplementary Table S2). We further performed the correlation analysis to explore the specific relationship of the 41 DE genes. The results showed the levels of positive or negative correlation between each two individual genes in the GSE58294 dataset (Figure 4).

### GO and KEGG enrichment analysis of the DE autophagy-related genes of GSE58294

**Figure 5:**
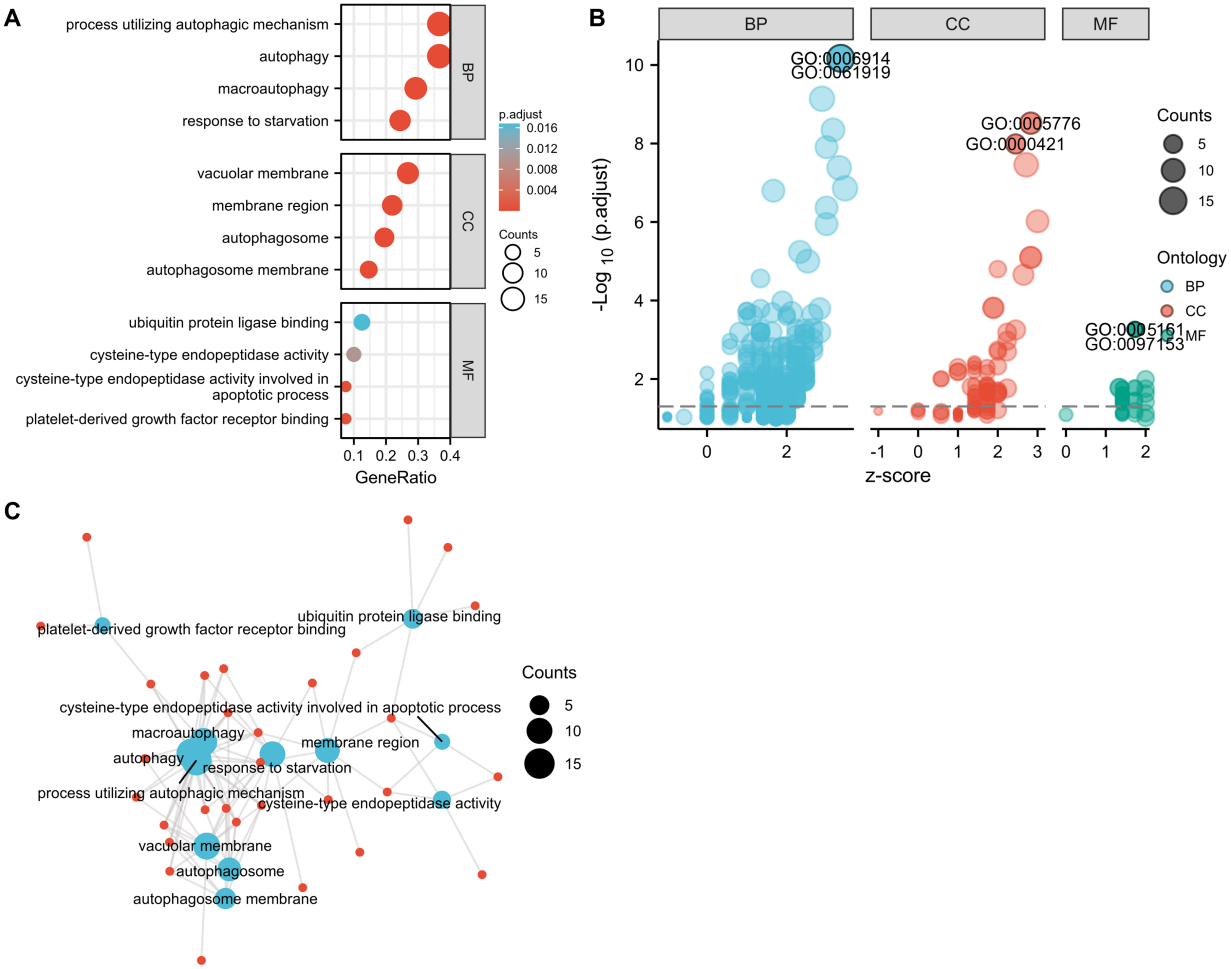
Gene ontology (GO) enrichment analysis of 41 differentially expressed autophagy-related genes. **(A)** The most significant enriched GO terms. **(B)** Bubble plot for the distribution of all enriched GO terms. **(C)** Connections between genes and GO enriched terms. Blue spot: GO term. Red spot: representative DE genes. BP, biological process; CC, cellular component; MF, molecular function.

**Figure 6:**
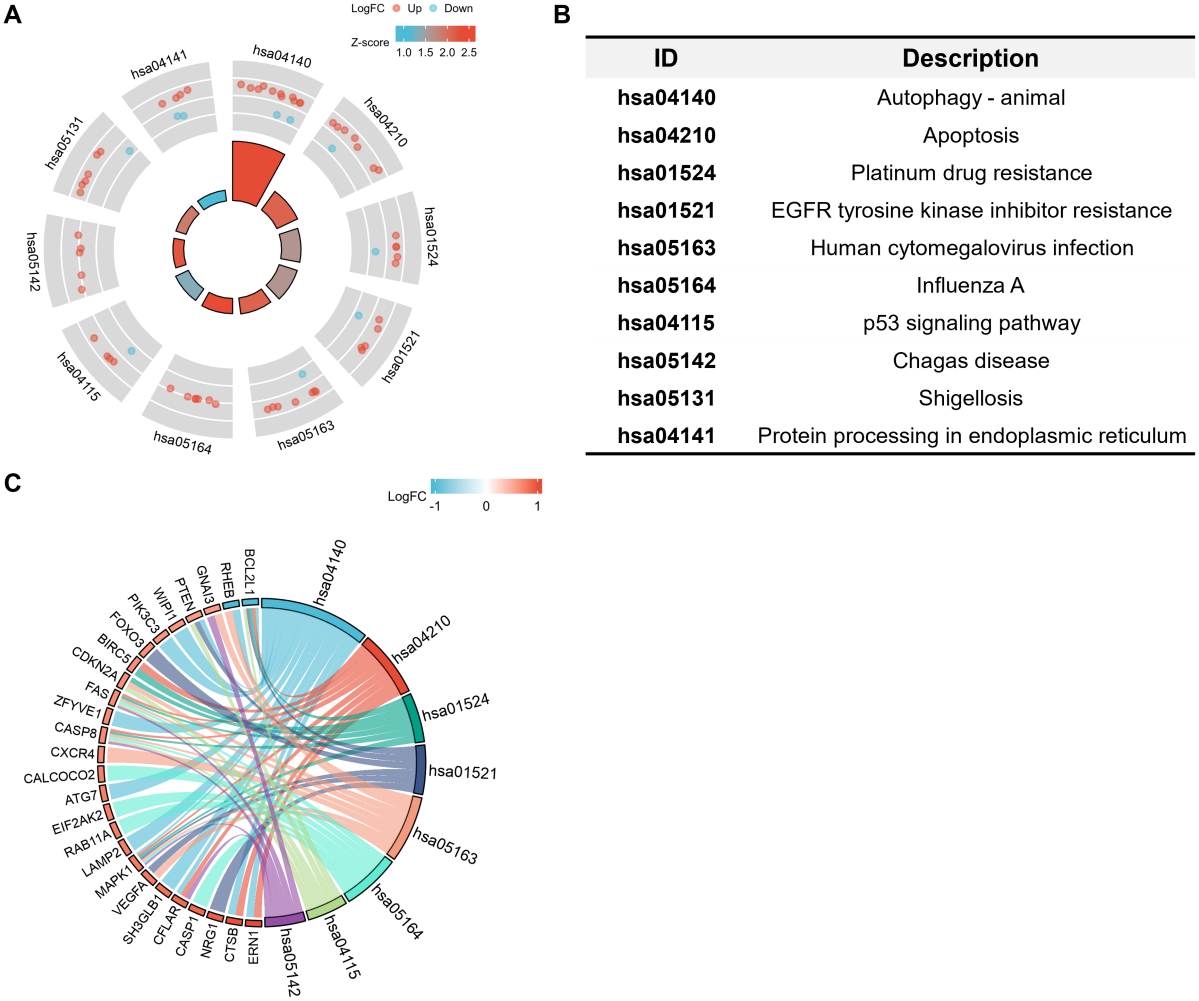
Kyoto Encyclopedia of Genes and Genomes (KEGG) analysis of 41 differentially expressed autophagy-related genes. **(A,B)** Statements of enriched KEGG pathways and distribution of the related DE genes. Red dots: upregulated DE genes. Blue dots: downregulated DE genes. The shade of the colors corresponds to the value of log_2_FC. The area of the central trapezoid corresponds to the counts of enriched DE genes in each pathway. **(C)** String diagram of the connections between main autophagy-related DE genes and the corresponded enriched KEGG pathways. The shade of colors of the small trapezoids besides the DE genes corresponds to the value of log_2_FC. The width of the cambered area beside the KEGG ID corresponds to the counts of enriched DE genes. Strings indicate the involvements of genes in the corresponded pathway. The diagrams represented the enriched pathways and involved DE genes with an adjusted value of *p* of <0.05.

The GO and KEGG enrichment analyses were conducted with R software to analyze the potential biological functions of the autophagy-related DE genes in CE stroke samples. The most significant enriched terms in GO include process utilizing autophagic mechanism, autophagy, macroautophagy, response to starvation (biological process), vacuolar membrane, membrane region, autophagosome, autophagosome membrane, ubiquitin protein ligase binding, cysteine-type endopeptidase activity, cysteine-type endopeptidase activity involved in the apoptotic process, and platelet-derived growth factor receptor binding (molecular function; Figures 5A,B; Supplementary Table S3). The interaction between each enriched GO term was exhibited (Figure 5C). In KEGG enrichment analysis, the autophagy-related DE genes in CE stroke samples are mainly involved in the process of the autophagy–animal pathway (Figures 6A,B; Supplementary Table S4). The other enriched KEGG pathways were apoptosis, platinum drug resistance, EGFR tyrosine kinase inhibitor resistance, human cytomegalovirus infection, influenza A, p53 signaling pathway, Chagas disease, Shigellosis, and protein processing in the endoplasmic reticulum (Figure 6B). Moreover, the most significantly expressed genes involved in the enriched KEGG pathways were identified and their interactions were shown with colorful strings (Figure 6C; Supplementary Table S4).

### Cross-validation of the autophagy-related DE genes with other GEO datasets (GSE66724, GSE41177, and GSE22255).

**Figure 7:**
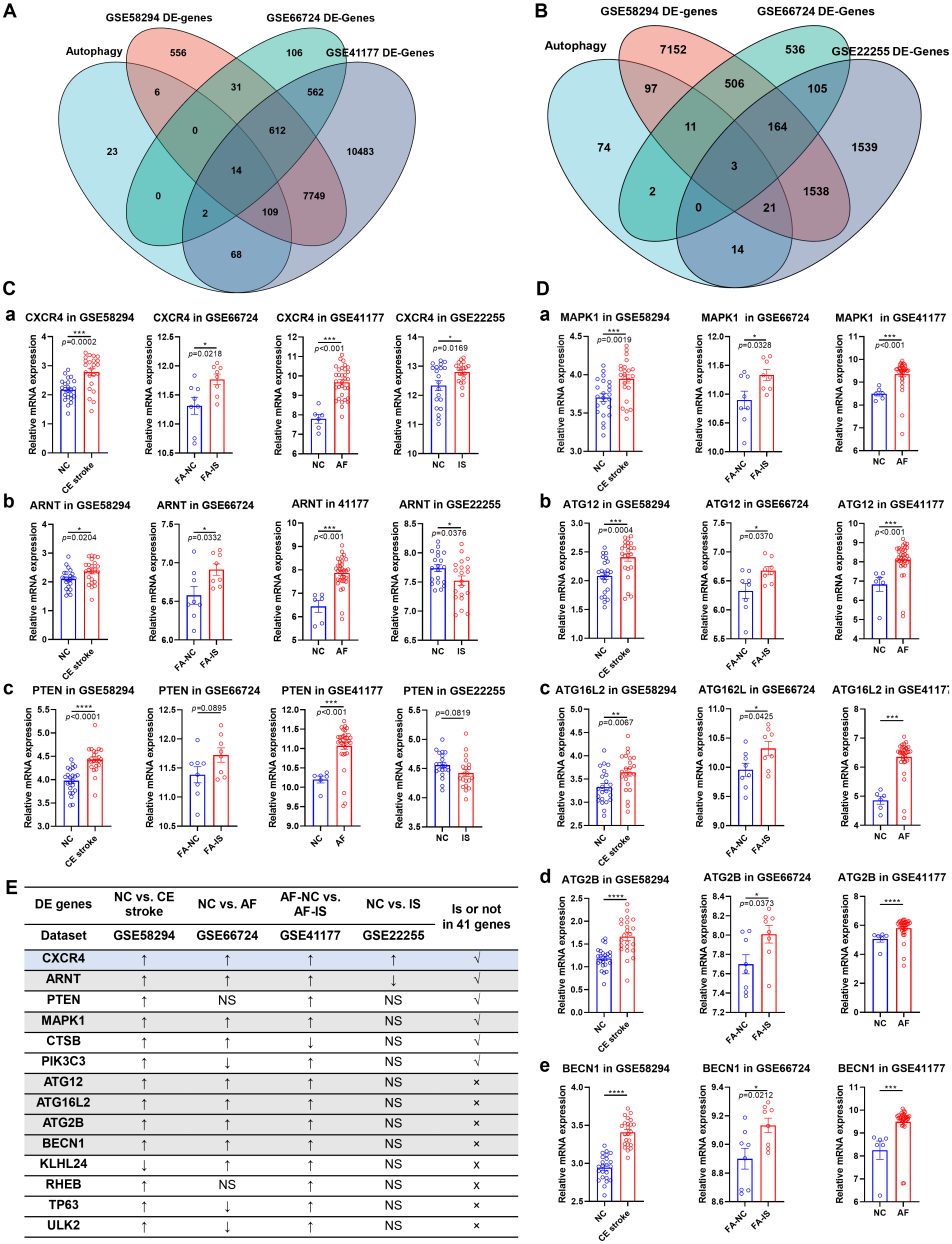
Cross-validation of the differentially expressed autophagy-related genes in CE stroke with GSE66724, GSE41177, and GSE22255 datasets. **(A)** Venn diagram of autophagy-related DE genes in GSE58294, GSE66724, and GSE41177. **(B)** Venn diagram of autophagy-related DE genes in GSE58294, GSE66724, and GSE22255. **(C)** Expression levels of CXCR4 **(a)**, ARNT **(b),** and PTEN **(c)** in four GO datasets. **(D)** Expression levels of MAPK1 **(a)**, ATG12 **(b)**, ATG16L2 **(c)**, ATG2B **(d)**, and BECN1 **(e)** in four GO datasets. All statistical analyses were performed with Student’s *t*-test. The significant differences were indicated with asterisks. ^*^p < 0.05, ^**^p < 0.01, ^***^p < 0.001, and ^****^p < 0.0001. **(E)** Summary of the differences for the 14 autophagy-related DE genes in four GO datasets. Changes in gene expression levels were indicated with arrows. ↑: Upregulated in samples of patients. ↓: Downregulated in samples of patients. NS, not significant.

To further identify the specific autophagy-related genes in CE stroke from other sources of strokes, the GSE66724, GSE41177, and GSE22255 datasets were downloaded for cross-validation with the GSE58294 dataset. Because AF is the most common cause of cardioembolic stroke (3, 20), the GSE66724 dataset was used to identify the hub genes of CE stroke from cardiac diseases, which includes transcriptomes of peripheral blood samples from eight patients with AF stroke and eight controls with atrial fibrillation. In addition, GSE41177 was introduced to further clarify the symbolic DE genes specialized in AF with stroke, which consists of 16 patients with persistent AF and three controls with sinus rhythm. The DE genes of GSE58294, GSE66724, and GSE41177 were screened in the autophagy gene hub and compared with each other. 14 autophagy-related hub genes were identified differentially expressed in all three datasets: ARNT, ATG12, ATG16L2, ATG2B, BECN1, CTSB, CXCR4, KLHL24, MAPK1, PIK3C3, PTEN, RHEB, TP63, and ULK2 (Figure 7A; Supplementary Table S5). To pick the particular DE genes in CE stroke, DE genes in all types of strokes were excluded by bringing in the GSE22255 dataset that consists of 20 ischemic (IS) patients and 20 sex- and age-matched controls. The DE genes of GSE58294, GSE66724, and GSE22255 were screened in the autophagy gene hub and compared with each other. A set of three differentially autophagy-related genes were identified: ARNT, CXCR4, and PTEN (Figure 7B; Supplementary Table S6). These three DE autophagy-related genes all happened to be included in the former identified 14 genes; therefore, the remaining 11 genes could be the potential autophagy-related DE genes, especially for CE stroke. To check whether the variations of these DE genes were consistent in each dataset, the expression levels of these genes were verified with Student’s t-test (Figures 7C,D). Among the 14 autophagy-related DE genes, although ARNT, CXCR4, and PTEN were identified to be differentially expressed in four datasets with the R package, only CXCR4 was upregulated in all datasets (Figure 7C). ARNT was consistently upregulated in GSE58294, GSE66724, and GSE41177 but downregulated in GSE22255. While PTEN was just significantly upregulated in GSE58294 and GSE41177 but not significantly changed in GSE66724 and GSE22255 (Figure 7C). In the remaining 11 genes which were differentially expressed in GSE58294, GSE66724, and GSE41177 but not in GSE22255 in the R program, five genes showed consistent variation that they were upregulated in the three datasets, including MAPK1, ATG12, ATG16L2, ATG2B, and BECN1 (Figure 7D). The rest genes showed inconsistent variations in the three datasets. CTSB, PIK3C3, and TP63 were upregulated in GSE58294 and GSE41177 but downregulated in GSE66724. RHEB and ULK2 were upregulated in GSE58294 and GSE41177 while not significantly changed in GSE66724. KLHL24 was downregulated in GSE58294 but upregulated in GSE66724 and GSE41177 (Supplementary Figure S1). The variations of genes are summarized in Figure 7E.

## Discussion

Cardioembolic strokes carry high morbidity and are associated with early recurrence in patients. Identification of hub genes in CE stroke is critically important to prevent the occurrence and relapses of the disease. Autophagy functions as an essential cellular process to maintain cellular homeostasis and organismal survival. Targeting autophagy turns out to be potential clinical therapeutics for the treatment of ischemic stroke (1). Although several autophagy-related genes were reported to be involved in ischemic stroke (21), the roles of autophagy-related genes have not been explored in CE stroke.

In this study, the 41 autophagy-related DE genes were for the first time identified in CE stroke through bioinformatics analysis. Moreover, several autophagy-related genes were identified as hub genes for CE stroke. Because animal models could hardly mimic the episodes of CE stroke, the specific functions of individual genes were barely studied. However, some of the autophagy-related genes have been previously investigated in other types of strokes. For example, the upregulation of neuronal RHEB (S16H) could significantly attenuate ischemic damage and behavioral impairments in a mouse model of photothromobosis-induced ischemic stroke (22), which was here found downregulated in the serum of CE stroke patients. Moreover, BAG1 (23) and USP10 (24) could protect against cerebral ischemia injury in ischemic animals, which were both downregulated in CE stroke serum samples in this study. The anti-apoptotic protein BCL2L1 binds with BECN1 as a suppressor of autophagy (25), while their expression changed in the opposite direction, indicating a more complicated situation of autophagy in CE stroke. For the upregulated autophagy-related genes in CE stroke patients, the heterozygous depletion of the autophagy adaptor protein WDFY3/ALFY significantly accelerates the age of onset and progression of HD (Huntington’s disease) pathogenesis, revealing the requirement of WDFY3 in clearing proteinaceous deposits (26) in the brain. The upregulation of WDFY3 in the serum of CE stroke patients indicated an elevated selective elimination of β-sheet misfolded aggregates. A common coding variant in SERPINA1 increases the risk for large artery atherosclerotic stroke (27, 28). The ER stress response has been well studied to aggravate the loss of neuronal function in stroke (29, 30), in which ERN1 plays a vital signal role. Inhibition of autophagy by ATG3 silencing activated the phosphoinositide 3-kinase (PI3K)/Akt pathway and attenuated inflammation in OGD/R-challenged brain microvascular endothelial cells (31), indicating that the ATG3-mediated autophagy might play a detrimental role in stroke. NRG-1 induces hypertrophy and worsens cardiac performance in post-myocardial infarction (MI) rats (32). Therefore, several autophagy-related DE genes that were upregulated in the serum of CE stroke patients were reported to be deleterious in ischemic models, supporting previous evidence that ischemic stroke usually evokes excessive autophagy (33, 34), and targeting autophagy dysfunction could attenuate cerebral ischemic stroke via mTOR/Ulk1 pathway in MCAO animals (35). However, overexpression of CFLAR could markedly alleviate cerebral I/R injury in MCAO mice by suppressing inflammation and ER stress (36), suggesting that variations of the autophagy-related DE genes might function to restore the damaged brain in CE stroke rather than the immediate ischemic injuries. The protective responses of autophagy to ischemic stroke have been previously reported (37). Therefore, the functions of DE genes in CE stroke need further studies and verification. We aim to discover more autophagy-related risk factors involved in CE stroke pathogenesis and find out the potential therapeutic targets for CE stroke treatment.

The potential biological functions of these autophagy-related DE genes were also conducted through GO and KEGG enrichment analysis. GO analysis of autophagy-related DE genes indicated several enriched terms related to the process utilizing autophagic mechanism and autophagy. In addition to the autophagy-related pathway, p53 signaling pathway, apoptosis, and protein processing in the endoplasmic reticulum (ER) were indicated in the KEGG enrichment. In addition to the autophagy-related pathway, p53 signaling pathway, apoptosis, and protein processing in the endoplasmic reticulum (ER) were indicated in the KEGG enrichment. Autophagy can be evoked by ischemia, while cerebral ischemia–reperfusion-induced autophagy could protect against neuronal injury by PARK2 involved mitochondrial clearance (38). Autophagy dysfunction played a role in ischemic stroke, and targeting autophagy could attenuate cerebral ischemic stroke in MCAO animals (35). As one of the regulated cell death mechanisms, apoptosis could mediate vascular and neural pathology in stroke (39). Elevated levels of FADD (Fas-associated protein with death domain) and caspase-8 were associated with an increased incidence of ischemic stroke (40). Amounts of molecules targeted apoptosis and cooperated with other biological processes to function in ischemic stroke. Under ischemic conditions, the NLRC4 inflammasome complex mediated the inflammatory response, as well as apoptotic and pyroptotic cell death of microglial cells (41). P53 signaling played a role in the activation of autophagy which inhibits tumorigenesis (42, 43) while its interaction with NFκB promoted cell apoptosis and neuronal deaths in ischemic injuries (44, 45). Reticulon Protein 1-C (RTN1-C) played an important role in ER stress, the knockdown of which could reverse ischemia-induced apoptosis and attenuate the vulnerability of OGD/R-treated neural cells (46). Further investigation will be necessary to explore the potential biological functions of these autophagy-related DE genes.

Based on the bioinformatics analysis of GSE58724, GSE66724, GSE41177, and GSE22255 datasets were introduced for cross-validation, and we got more particular knowledge about these autophagy-related genes. Among the 14 autophagy-related DE genes, only CXCR4 (stromal cell-derived factor-1α/cysteine-X-cysteine chemokine receptor 4) was upregulated in all datasets with further validation by Student’s t-test (Figure 7C) and, therefore, identified as a hub gene for all types of strokes. CXCR4 promoted initial monocyte infiltration and subsequent territorial restriction of monocyte-derived macrophages to infarct tissue in the stroke brain. CXCR4 deficiency could reduce monocyte infiltration and blunt the expression of pattern recognition and defense response genes in monocyte-derived macrophages after transient focal ischemia (47). The increase of CXCR4 might indicate an accumulation of inflammatory reactions in human blood as an early warning for ischemia. Consistently, the CXCR4 antagonists AMD3100 and CX549 were both efficient to mobilize bone marrow hematopoietic stem cells (HSCs), improving behavioral performances, reducing brain infarction, and suppressing the expression of inflammatory markers in stroke brain (48, 49). Therefore, targeting the inhibition of CXCR4 could prevent the occurrence of stroke.

ARNT, MAPK1, ATG12, ATG16L2, ATG2B, and BECN1 were consistently upregulated in GSE58294, GSE66724, and GSE41177 but not in GSE22255, thus counting as hub genes especially for CE stroke to distinguish CE stroke risk from cardiogenic diseases, in which ARNT and MAPK1 belong to the identified 41 autophagy-related DE genes.

Aryl hydrocarbon receptor nuclear translocator (ARNT) is also called hypoxia-inducible factor-1beta (HIF-1β), which is upregulated in the serum of CE stroke patients. As the principal transcription factor is activated by low oxygen tensions, HIF-1 is essential for mammalian development. HIF-1β mRNA and protein were directly regulated by NF-κB and its overexpression could rescue HIF-2α protein levels following NF-κB depletion (50). HIF-1β was selectively repressed by IFN-γ in a JAK-dependent manner, indicating its vital role in inflammation (51). In humans, the levels of HIF-1 transcripts are directly correlated with those of hypothalamic transcripts for proteins involved in inflammation, regulation of apoptosis, autophagy, and the ubiquitin/proteasome system; in mice with high fat diet, diet-induced obesity was accompanied by increased HIF-1 expression (52). Moreover, mice carrying the myeloid HIF-1β deletion displayed aggravated hypoxic phenotypes and significantly greater weight loss and significantly higher cardiac index values than the wild-type group (53). Considering that platelet thrombus formation is usually positively correlated with hyperlipidemia, the increase of HIF-1β might indicate the accumulated inflammation and the disorder of lipid metabolism in CE stroke.

MAPK1 is an essential component of the MAP kinase signal transduction pathway. The MAPK/ERK cascade was mostly studied in cancer. It plays a role in the initiation and regulation of meiosis, mitosis, and postmitotic functions in differentiated cells by phosphorylating several transcription factors (54). MAPK phosphorylated PML nuclear body scaffold and promoted its interaction with peptidylprolyl cis/trans isomerase, NIMA-interacting 1 (PIN1), leading to PML degradation. While PIN1 could facilitate the stability of Notch1 intracellular domain (NICD1) and promote the proapoptotic function of NICD1 following ischemic stroke (55). Overexpression of miR-194-5p protected against myocardial ischemia/reperfusion injury by negatively regulating MAPK1, which involved the inhibition of cardiac apoptosis and oxidative stress (56). The research indicates that the increase of MAPK1 contributes to abundant apoptosis in ischemia-induced injury. As to the role of MAPK1 in autophagy, the activation of MAPK1/3 was reported to ameliorate liver steatosis and increase the autophagic flux and ATG7 (autophagy-related 7) levels. Moreover, ATG7 knockdown reversed the ameliorated liver steatosis in MAPK1/3-activated db/db mice (57), suggesting that MAPK1 participates in autophagy in an ATG7-dependent approach. ATG7 was also identified as a DE gene in CE stroke (GSE58294) but not in generalized stroke (GSE22255). ATG7 encodes an E1-like activating enzyme thus involving two ubiquitin-like systems required for autophagy. ATG7 facilitated LC3-I lipidation with phosphatidylethanolamine to form LC3-II which is found on autophagosomal membranes and its disorder leads to poor or absent autophagic function (58). With triggering lysosomal membrane permeabilization, ATG7 facilitated the induction of apoptosis after lysosomal photodamage (59). Therefore, the increase of MAPK1 in the serum of CE stroke patients might indicate the disrupted homeostasis of the ubiquitin system and more serious impairments of autophagosome-lysosomal functioning and possibly the following apoptosis.

The remaining autophagy-related genes, such as ATG12, ATG16L2, ATG2B, and BECN1 were also significantly increased in CE strokes while not all types of strokes. These genes all directly participate in the autophagy process where bulk protein degradation happens. Cytoplasmic components, including organelles, are enclosed in autophagosomes and delivered to lysosomes or vacuoles for degradation. As the ubiquitin-like protein involved in autophagy vesicles formation, ATG12 conjugated with ATG5 through a ubiquitin-like conjugating system involving ATG7 and ATG10 to deliver its function (60). ATG12-mediated autophagy was reported to interact with miR-26a-5p in ischemia reperfusion (61). The Atg5–Atg12 conjugation was reported to damage the type I IFN production pathway thus negatively regulating the innate antiviral immune response (62), indicating a role as an inflammatory mediator. Autophagy-related 16 like 2 (ATG16L2) is a critical autophagy-related gene necessary for autophagosome formation. Knockdown of ATG16L could reduce NF-κB degradation and excrete inflammatory cytokines (63). ATG16L2 could inhibit NLRP3 inflammasome activation (64), indicating its protective role in inflammatory diseases. The implication of ATG12 and ATG16L2 in the inhibition of inflammation might indicate a reduction of inflammatory responses in CE stroke, which was involved with autophagy. Autophagy-related protein 2 homolog B (ATG2B) was required for both autophagosome formation and regulation (65). It was a downstream target of miR-130a, and the pathway was involved in LncRNA-HOTAIR activating autophagy (66). By targeting ATG2B to inhibit autophagy, miR-130a could attenuate the proliferation of vascular smooth muscle cells, and the increased expression of ATG2B and LC3 II/I was observed in the artery vascular tissues of atherosclerosis obliterans patients (67), suggesting that ATG2B might contribute to impairing myocardial function thus promoting the occurrence of CE stroke. BECN1 played a central role in autophagy (68), which acted as a core subunit of the PI3K complex, and the different complex forms might play a role in multiple membrane trafficking pathways (68).

Although the functions of the identified hub genes were barely studied in CE stroke, their critical roles in autophagy were obvious. The upregulation of these genes indicates the drastic autophagy process evoked in CE stroke. However, as a double sword in the cellular adaptive system, autophagy delivers different roles in regulating neuronal deaths in stroke. Therefore, we ought to take a closer look at the specific functions of autophagy DE genes in CE stroke for that they were identified from various sources of GEO datasets. Under normal conditions, the autophagy process is activated to help with cell survival by regulating the clearance and re-use of intracellular constituents, which is considered the cellular adaptive response to stress. However, persistent stress would induce excessive autophagy, which exceeds the cellular adaptive capacity and then facilitates necrosis and apoptosis thus leading to cell death (69). In the case of ischemic stroke, when the cerebral blood flow is tremendously interrupted over a few seconds, neurons and glial cells responded to the stress immediately due to oxygen and glucose deprivation. Once the ischemia lasts for several minutes, neuronal deaths start from the core infarct region following irreversible neural injuries (70). Autophagy activation after ischemic insult is only transient as a prelude to a prolonged dysfunction of autophagic machinery (71). Long before 3-methyladenine (3-MA, a selective autophagy inhibitor) has been reported to strongly reduce neuronal autophagy and the infarct volume at >4 h after the beginning of ischemia in rats with transient focal cerebral ischemia (72). For the samples analyzed in GSE58294, the peripheral bloods were collected from CE stroke patients without any intervention in less than 3 h since the occurrence of CE stroke. At this point, lacking in glucose and oxygen has brought out severe brain damage; therefore, disordered autophagy is more likely to contribute to cell deaths. Through the broken blood–brain barrier, the expression of autophagy-related DE genes in peripheral bloods might change synchronously with the variations in the central nervous system upon stroke. By introducing GSE66724, the autophagy-related DE genes in CE stroke were further identified from the AF cohorts. For these sources, the AF-IS patients suffered CE stroke between 2 and 11 years previously; therefore, the samples were collected after recovery from stroke. The identified autophagy DE genes indicate the increased autophagy occurring in the blood of AF-IS patients. It could be the adaptive self-cleaning mechanism after suffering a stroke, or it might reflect an abnormal physiological state of AF patients risking stroke. Taken together, whatever the situation is, the identified autophagy-related hub genes indicate the dysfunction of autophagy in biological processes, which would be assumed to be potential risk factors for stroke for patients carrying cardiovascular and cerebrovascular diseases.

There exist some limitations in this research. First, expression levels of the identified genes were not validated in blood samples of CE stroke patients by quantitative tests due to the difficulty in collecting blood samples from CE stroke patients without any intervention within 3 h. These procedures cannot be ethically approved currently. However, the verification was carried out between GEO datasets to pick the hub genes for CE stroke. Second, the number of clinical samples included in this study is limited, especially the GSE66724 dataset. With larger cohorts of patients, we will be able to identify a more accurate hub gene. Third, although several hub genes for CE stroke were screened from the autophagy-related differentially genes, the specific mechanisms of these genes were not explored *in vitro* or *in vivo* ischemia models. We would undertake further studies to demonstrate their roles in CE stroke in the future.

In conclusion, 41 autophagy-related DE genes were identified in CE stroke. In addition, the interactions between the hub genes were analyzed, as well as their involved pathways and biological processes. Moreover, by cross-validation with other CE stroke-related datasets, CXCR4 was identified as a pathogenetic marker for all types of strokes. ARNT, MAPK1, ATG12, ATG16L2, ATG2B, and BECN1 were identified as particular pathological markers for CE stroke while not other types of strokes, in which ARNT and MAPK1 belong to the 41 hub genes. The abnormity of these potential risk genes could provide an early warning of stroke for patients carrying cardiogenic diseases. Moreover, the identified hub genes offered potential therapeutic targets for the treatment of CE stroke. However, the interactions between these genes and other factors (e.g., lncRNA, miRNA, and other genes) predict their intricate roles in the organisms, waiting to be further discovered.

## Data availability statement

Publicly available datasets were analyzed in this study. This data can be found at: https://www.ncbi.nlm.nih.gov/geo/query/acc.cgi. The accession numbers are: GSE58294; GSE66724; GSE41177; and GSE22255.

## Author contributions

YY, MZ, and ZL conducted most of the data analysis and contributed to the manuscript preparation. SH, ZL, and XR helped with data analysis. LW instructed the writing of the manuscript. ZW supervised the study. SS designed and supervised the study. All authors contributed to the article and approved the submitted version.

## Funding

This study was supported by grants from the National Natural Science Foundation of China (81774388 to SS, 82104640 to YY, 81873029 to ZW, 81901371 to SH, and 82004465 to LW), Shanghai Sailing Program (no. 19YF1448500 to YY), and Shanghai Mental Health Center Grant (no. 2022zd02 to HS).

## Conflict of interest

The authors declare that the research was conducted in the absence of any commercial or financial relationships that could be construed as a potential conflict of interest.

## Publisher’s note

All claims expressed in this article are solely those of the authors and do not necessarily represent those of their affiliated organizations, or those of the publisher, the editors and the reviewers. Any product that may be evaluated in this article, or claim that may be made by its manufacturer, is not guaranteed or endorsed by the publisher.

## Footnotes

^1^ http://www.autophagy.lu/index.html

^2^ http://www.ncbi.nlm.nih.gov/geo/

^3^ https://string-db.org/

## Supplementary material

### SUPPLEMENTARY TABLE S1

The differentially expressed autophagy-related genes in stroke samples compared to healthy samples based on GSE58294.

### SUPPLEMENTARY TABLE S2

The interactions between hub genes in String map based on GSE58294.

### SUPPLEMENTARY TABLE S3

The enrichment of GO term of the hub genes based on GSE58294.

### SUPPLEMENTARY TABLE S4

The enrichment of KEGG pathway of the hub genes based on GSE58294.

### SUPPLEMENTARY TABLE S5

Venn for GSE66724-GSE41177-GSE58294-autophagy datasets.

### SUPPLEMENTARY TABLE S6

Venn for GSE66724 - GSE22255 -GSE58294-autophagy datasets.

### SUPPLEMENTARY TABLE S7

Information of samples analyzed in all GEO datasets.

### SUPPLEMENTARY FIGURE S1

Expression levels of CTSB **(A)**, PIK3C3 **(B)**, TP63 **(C)**, RHEB **(D)**, ULK2 **(E)** and KLHL24 **(F)** in the 4 GEO datasets. All statistical analyses were performed with Student’s *t* test. The significant differences were indicated with asterisks. **p*<0.05, ***p*<0.01, ****p*<0.001, *****p*<0.0001.
