# Supplementary material for: Identification and cross-validation of autophagy-related genes in cardioembolic stroke
Source: Front Neurol. 2023 May 25;14:1097623. doi: 10.3389/fneur.2023.1097623 (PMC10248509; doi:10.3389/fneur.2023.1097623)
Supplement: Supplementary file 8 [file Image_1.pdf]

Supplementary figures and figure legends

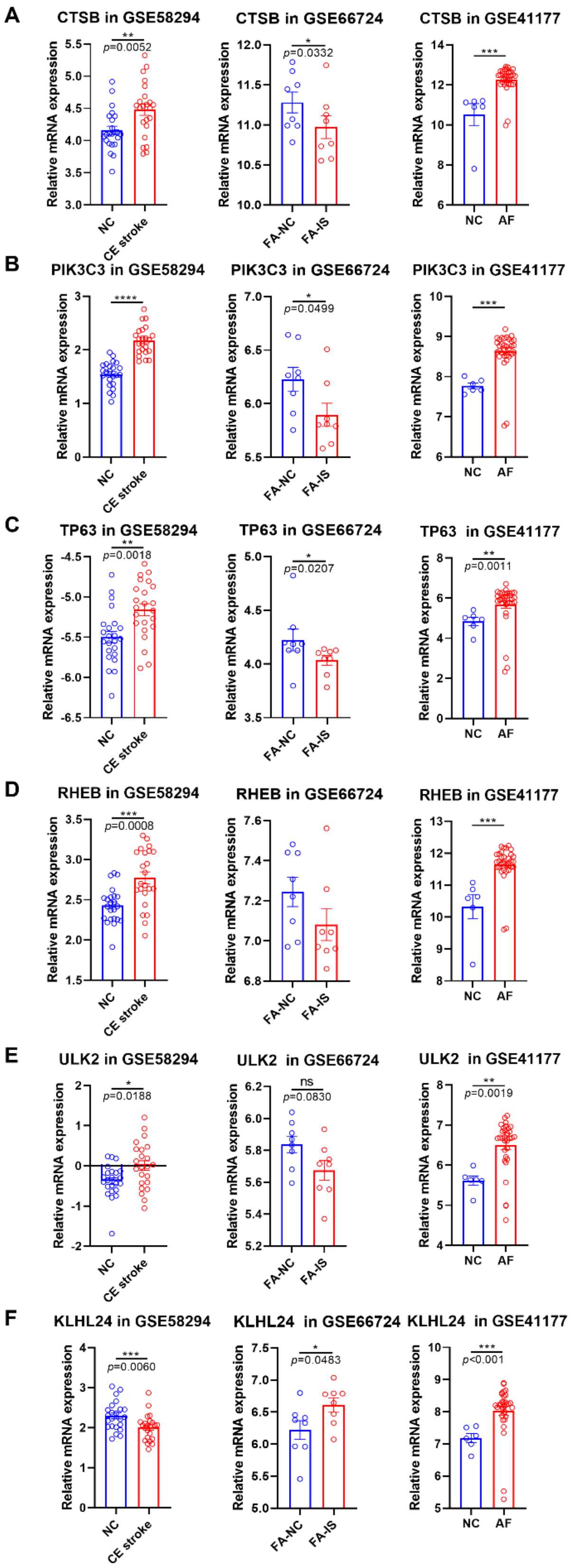

Figure S1. Expression levels of CTSB (A), PIK3C3 (B), TP63 (C), RHEB(D), ULK2 (E) and

KLHL24 (F) in the 4 GEO datasets. All statistical analyses were performed with Student's t test. The significant differences were indicated with asterisks. * $p<0.05$, ** $p<0.01$, *** $p<0.001$, **** $p<0.0001$
